# Revisiting the bi-factor structure of the short version of the Warwick–Edinburgh mental well-being scale with samples in China

**DOI:** 10.3389/fpsyg.2025.1422065

**Published:** 2025-02-24

**Authors:** Sai-fu Fung, Guang Zeng, Ho-luan Hermes Chuang, Sin-man Li, Jingwen Lee, Jonathan Chun Luen Tse

**Affiliations:** ^1^Department of Social and Behavioural Sciences, City University of Hong Kong, Kowloon, Hong Kong SAR, China; ^2^School of Psychology, South China Normal University, Guangzhou, China; ^3^School of Philosophy, Psychology, and Language Sciences, The University of Edinburgh, Edinburgh, United Kingdom

**Keywords:** bi-factor structure, confirmatory factor analysis, mental well-being, student, SWEMWBS, WEMWBS, women

## Abstract

**Aim:**

This paper aims to evaluate the factor structure and psychometric properties of the Short Warwick-Edinburgh Mental Well-being Scale (SWEMWBS) based on Chinese samples recruited from secondary schools, universities, and workplaces.

**Methods:**

The psychometric properties of the SWEMWBS were evaluated in four cross-sectional studies with a total of 1,729 respondents from Hong Kong and Chinese mainland. Criterion validity was assessed using well-established measures of well-being, affect, and life satisfaction (PANAS, WHO-5, and SWLS). Confirmatory factor analysis was employed to evaluate the unidimensional and bi-factor structure of the SWEMWBS.

**Results:**

The results indicated that both the Traditional and Simplified Chinese versions of the SWEMWBS possess good psychometric properties, with coefficient alpha and omega exceeding the acceptable range. Both exploratory factor analysis and confirmatory factor analysis suggested that the items are suitable for forming a unidimensional scale. However, the bi-factor structure proposed in the recent literature contained some problematic items that yielded negative and weak factor loadings.

**Conclusion:**

The unidimensional SWEMWBS is applicable to respondents from diverse socio-economic and cultural backgrounds. The validated Traditional and Simplified Chinese versions of the SWEMWBS provide researchers and clinical practitioners with a reliable and convenient measure of mental well-being for use in Chinese contexts.

## 1 Introduction

The recent pandemic and global public health crisis presented an unprecedented stressor to patients and healthcare systems worldwide, affecting not only physical health but also mental health. Numerous epistemological and clinical studies have examined the impact of the health crisis on mental well-being among students (Paton et al., 2023), the general public (Gül and Yesiltas, 2022), and medical professionals (Aruta et al., 2023; Laker et al., 2023; Yeh et al., 2022). There is also a significant body of literature demonstrating the close relationship between mental health and employment, productivity, criminal activity, prosocial behaviour, and education (Maccagnan et al., 2019). Mental health has received increased attention within the literature, specifically when referring to subjective well-being and functioning well in hedonic and eudaimonic areas (Forgeard et al., 2011; Huppert and So, 2013; McQuaid and Kern, 2017). Nonetheless, there is a call for the development and validation of screening tools and diagnostic instruments with good psychometric properties to assess mental well-being in different cultural settings (Ransing et al., 2020). Up-to-date measures of positive mental health and well-being are still lacking, particularly in the Chinese context (Fung, 2019).

Baumgardner (2009) emphasised the need to develop an empirically based conceptual understanding and language for describing healthy human functioning that parallels the classification and understanding of mental health disorders. In response, the Warwick-Edinburgh Mental Well-being Scale (WEMWBS) was constructed to measure mental well-being with a specific focus on hedonic and eudaimonic aspects (Tennant et al., 2007). The WEMWBS has been translated into over 30 languages and is used worldwide in countries such as Brunei (Rahman et al., 2023), Denmark (Hauch et al., 2023), Finland (Sarasjärvi et al., 2023), France (Barbalat et al., 2023), Spain (Castellví et al., 2014), and Sri Lanka (Perera et al., 2022).

The WEMWBS measures two major areas of positive mental health: the hedonic and eudaimonic perspectives. The hedonic perspective focuses on the subjective experience of happiness and life satisfaction, while the eudaimonic perspective emphasises psychological functioning and self-realisation (Tennant et al., 2007). The instrument was developed to capture a broad conception of well-being, including affective-emotional aspects, cognitive-evaluative dimensions, and psychological functioning (Tennant et al., 2007). To address potential gender bias, a shortened version of the scale, the SWEMWBS, was developed, reducing the number of scale items from 14 to 7. According to the original scale developers and recent validation studies, the abbreviated version demonstrates comparable psychometric properties to the full 14-item version (Fung, 2019; Stewart-Brown et al., 2009). The SWEMWBS has been validated in various Chinese contexts, including ethnic Chinese minorities in the UK (Taggart et al., 2013), medical professionals, patients, and university students in China (Dong et al., 2016; Dong et al., 2019; Fung, 2019), and patients hospitalised with mental health disorders in Hong Kong (Ng et al., 2014), as well as the general population in Hong Kong (Sun et al., 2019).

However, the dimensionality and item composition of the SWEMWBS have been questioned in recent years (Lang and Bachinger, 2017; Ringdal et al., 2018). The SWEMWBS has been found to have a one-dimensional factor structure among Danish schoolchildren (Hauch et al., 2023), adolescents in the United Kingdom (Hanzlová and Lynn, 2023), and Norwegian and Swedish adults (Haver et al., 2015). In a SWEMWBS study conducted in Chinese mainland, Hong et al. (2023) also found a single-factor model, despite the fact that it was an online survey, making it difficult to be replicated without a probability sample. However, in a study of Finnish adults, Sarasjärvi et al. (2023) found that a bi-factor model best fitted for the factor structures, which includes all seven items related to mental well-being and an additional latent factor named eudaimonic well-being. This demonstrates better psychometric properties than the original unidimensional structure. To address this controversy, the present study has two main objectives. First, it aims to further evaluate the dimensionality and psychometric properties of the SWEMWBS with additional empirical evidence. Second, the study aims to validate the SWEMWBS in a variety of populations, including adolescents, university students, and female working adults in Hong Kong and Chinese mainland. While the Simplified Chinese version has been validated in both medical and non-medical populations in China (Dong et al., 2016; Dong et al., 2019; Fung, 2019), it is worthwhile to replicate the findings with more diverse samples. Furthermore, the Traditional Chinese version of the SWEMWBS has only been applied to clinical and medical-related populations in Hong Kong (Ng et al., 2014). Importantly, existing studies have only examined the internal consistency of the scale without using widely recognised validation methods, such as confirmatory factor analysis, to examine the scale’s construct validity (Hair, 2010; Jebb et al., 2021; Loewenthal, 2001).

Therefore, this study aims to fill this research gap by using the latest psychometric evaluation methods to provide a more holistic assessment of the dimensionality and psychometric properties of the SWEMWBS. The study will also contribute to the evaluation of both Chinese versions in different Chinese settings and cultures. Specifically, Traditional Chinese is primarily used in Hong Kong and among some overseas Chinese diaspora, while Simplified Chinese is the official language in Chinese mainland.

## 2 Methods

### 2.1 Participants

This paper reports the empirical findings from four cross-sectional studies involving a diverse population of working women, adolescents, and university students in Hong Kong and Chinese mainland. There are two criteria for inclusion: geographical location and language proficiency. Participants from Hong Kong (Studies 1 and 2) are required to be proficient in Traditional Chinese, while those recruited from Chinese mainland (Studies 3 and 4) must understand Simplified Chinese. Furthermore, all participants should be willing to provide informed consent, and individuals under 18 years old need to obtain additional informed consent from their parents or guardians.

Study 1 was conducted from February to April 2019 using online university intranet systems to recruit 148 young adults aged 18–25. Of these participants, 58.1% were male and 41.9% were female, studying at government-funded universities in Hong Kong.

Study 2, conducted over the same period, recruited 183 female working adults. The recruited sample of this study primarily consists of individuals aged 19–29, accounting for 35% of participants, followed by those aged 50–59 at 25.7%, 40–49 at 23.5%, 30–39 at 13.1%, and the smallest group, those aged 60 or above, at 2.7%. Among them, 31.1% held a college-level educational qualification or higher. The participants were recruited from a women’s organisation, family service centre, and community centre in Tin Shui Wai, in the northwest of Hong Kong.

Study 3 replicated Fung (2019) and was conducted from April to May 2019 with a sample of 511 college students recruited in Guangzhou, China. In this study, the original construal-related measures, such as positive and negative aspects of affect (PANAS), Five Well-Being Index (WHO-5), and Satisfaction with Life Scale (SWLS), were employed from the WEMWBS scale developers (Tennant et al., 2007) to evaluate the criterion validity of the scale. The average age of the respondents was 20.41 years, and 85.5% were female. Among them, 75.1% were from nuclear families, 20.4% were from extended families, and 4.5% had other family arrangements. Additionally, 80.6% of the parents of these college students were married.

Study 4 recruited 887 adolescents predominantly from three junior high schools in Tianjin City, China. Of these participants, 51.9% were male and 48.1% were female, with ages ranging from 11 to 15 years and a mean age of 13.6 years (SD = 0.86). Parental consent was obtained for these participants as they are underage. The study adhered to international ethical standards and was approved by the university’s ethics committee.

### 2.2 Measures

The SWEMWBS is a 7-item scale that measures positive affect, psychological functioning, and interpersonal relationships over the past 2 weeks (Stewart-Brown et al., 2009; Tennant et al., 2007). Each item is scored on a 5-point Likert scale from 1 = *none of the time* to 5 = *all of the time*. We adopted both Chinese versions of the SWEMWBS from previous validation studies. The Traditional Chinese version of the SWEMWBS used in Studies 1 and 2 was previously validated in patients with mental health disorders in a Hong Kong public hospital (Ng et al., 2014), while the Simplified Chinese version used in Studies 3 and 4 was based on a recent validation study among college students in Guangdong, southern China (Fung, 2019). According to Fung (2019), “traditional and simplified Chinese characters have significantly different visual-orthographic and topological properties, which affect their expression and usage.” Hence, this study used the Traditional Chinese version of the scale in Studies 1 and 2 in Hong Kong, whereas Studies 3 and 4 adopted the Simplified Chinese version to evaluate the scale’s psychometric properties with respondents in Chinese mainland.

The criterion validity of the SWEMWBS was evaluated through the pattern of correlations with other construal-related scales related to well-being, affect, and life satisfaction (Tennant et al., 2007). The World Health Organisation - Five Well-Being Index (WHO-5) (Bech, 2004, 2012; Bech et al., 2003) consists of 5 items rated on a six-point Likert-type scale, ranging from 1 (*at no time*) to 6 (*all of the time*). The Chinese version of the WHO-5 has been validated by Du et al. (2023).

The Positive and Negative Affect Schedule (PANAS) developed by Watson et al. (1988) includes two 10-items scales to measure positive and negative affect. The items are measured on a 5-point Likert-type scale, ranging from 1 (*not at all*) to 5 (*very much*). Scores for both positive and negative affect can vary from 10 to 50. Lower scores indicate reduced levels of positive or negative affect, while higher scores signify increased levels of positive or negative affect. The PANAS has been widely validated and utilised in different Chinese contexts (Chen et al., 2019; Kim and Wang, 2022; Song et al., 2024; Tu and Yang, 2016).

For life satisfaction, the Satisfaction with Life Scale (SWLS) (Diener et al., 1985) was used with the Chinese adapted version by Wang et al. (2017). The SWLS items are rated on a 7-point Likert-type scale, ranging from 1 (*strongly disagree*) to 7 (*strongly agree*).

### 2.3 Procedure

The internal consistency of the SWEMWBS was evaluated using Cronbach’s alpha (Cronbach, 1951) by examining the correlations and corrected item-total correlations between the seven items (Hair, 2010; Tabachnick, 2013). Additionally, coefficient omega, which is based on a one-factor model and provides a reliability estimate that overcomes the deficiencies of alpha, was also calculated (McDonald, 1999).

To assess the factor structure, a satisfactory factor structure was indicated by a Kaiser-Meyer-Olkin (KMO) value over 0.70 and Bartlett’s test of sphericity significant at *p* < 0.01 (Field, 2018). Exploratory factor analysis with maximum likelihood estimation was conducted using only the data from Study 1 to avoid the potential problem of overfitting (Fokkema and Greiff, 2017). Construct validity was evaluated using confirmatory factor analysis with maximum likelihood with mean- and variance-adjusted likelihood ratio test (MLMV), which has been suggested to provide better results in recent literature (Gao et al., 2020; Maydeu-Olivares, 2017). The criteria for model fit were: Comparative Fit Index (CFI) > 0.95, Tucker-Lewis Index (TLI) > 0.95, Root Mean Square Error of Approximation (RMSEA) < 0.06, standardised Root Mean Square Residual (SRMR) < 0.08 (Brown, 2014; Hair, 2010; Hu and Bentler, 1999; Schreiber et al., 2006). Additionally, *χ*^2^/df ≤ 3 was also considered indicative of good model fit (Bentler and Bonett, 1980; Byrne, 1998; Kline, 2005; Satorra and Bentler, 2001).

The above analyses were implemented using SPSS 28.0, R 4.3.1 computing language with the lavaan package version 0.6–16 (Rosseel, 2012), and MPlus 8.8 (Muthén and Muthén, 2017).

## 3 Results

### 3.1 Internal consistency

The results showed that the SWEMWBS had good internal consistency in both Study 1 (*n* = 148) and Study 2 (*n* = 183), with Cronbach’s alpha values above the acceptable range: 0.905 and 0.750, respectively. The SWEMWBS mean score was computed according to the instructions of the scale developers (Stewart-Brown et al., 2009). The mean score for the Traditional Chinese samples was 18.94 (SD = 3.335) and *a =* 0.860 (*n* = 331). The coefficient omega results for Study 1 (*n* = 148) also suggested that the SWEMWBS has good reliability with a *ω* value of 0.910 (Dunn et al., 2014; Lance et al., 2006; Nunnally and Bernstein, 1994). Table 1 presents the descriptive statistics and item correlations for all of the scale items from the combined samples for Studies 1 and 2 (*n* = 331). All of the item correlations (both *r* and *rs*) and corrected item-to-total correlations were over 0.350, which suggests that it is appropriate to combine the items for scale construction.

**Table 1:** Descriptive statistics and items correlations for the SWEMWBS.

Item	(1)	(2)	(3)	(6)	(7)	(9)	(11)
WEMWBS1	–	0.516	0.505	0.427	0.372	0.413	0.407
WEMWBS2	0.476	–	0.539	0.515	0.553	0.506	0.477
WEMWBS3	0.478	0.490	–	0.443	0.467	0.458	0.436
WEMWBS6	0.409	0.508	0.397	–	0.467	0.500	0.507
WEMWBS7	0.363	0.530	0.440	0.462	–	0.428	0.472
WEMWBS9	0.386	0.487	0.437	0.474	0.409	-	0.435
WEMWBS11	0.375	0.466	0.409	0.475	0.457	0.418	–
Mean	2.79	2.85	2.77	2.89	2.89	2.85	2.94
SD	0.891	0.886	0.853	0.845	0.874	0.926	0.911
Skewness	0.338	0.359	0.334	0.372	0.378	0.334	0.114
Kurtosis	−0.131	−0.579	−0.685	−0.541	−0.747	1.062	−0.707
r*_it_*	0.584	0.704	0.637	0.641	0.614	0.609	0.607
*a_iid_*	0.847	0.830	0.839	0.839	0.842	0.843	0.844

All correlations are significant at the 0.001 level (2-tailed); Lower triangle for Spearman correlations; upper triangle for Pearson correlations; r_it_ = Corrected item-total correlations; a_iid_ = Cronbach’s alpha, if item deleted.

### 3.2 Construct validity

The factor analysis results by principal components factor analysis with varimax rotation for Study 1 dataset (*n* = 148) also suggested that the SWEMWBS has an appropriate factor structure with a KMO value of 0.902 and Bartlett’s test of sphericity, *χ*^2^ = 589.920 (*p* < 0.001). The exploratory factor analysis also replicate the unidimensional factor structure of the SWEMWBS: the seven items loaded on a single factor, with loadings ranging from 0.697 to 0.805, which explained 58.296% of the total variance, confirming that the Chinese version measured the same construct as the English version.

**Figure 1:**
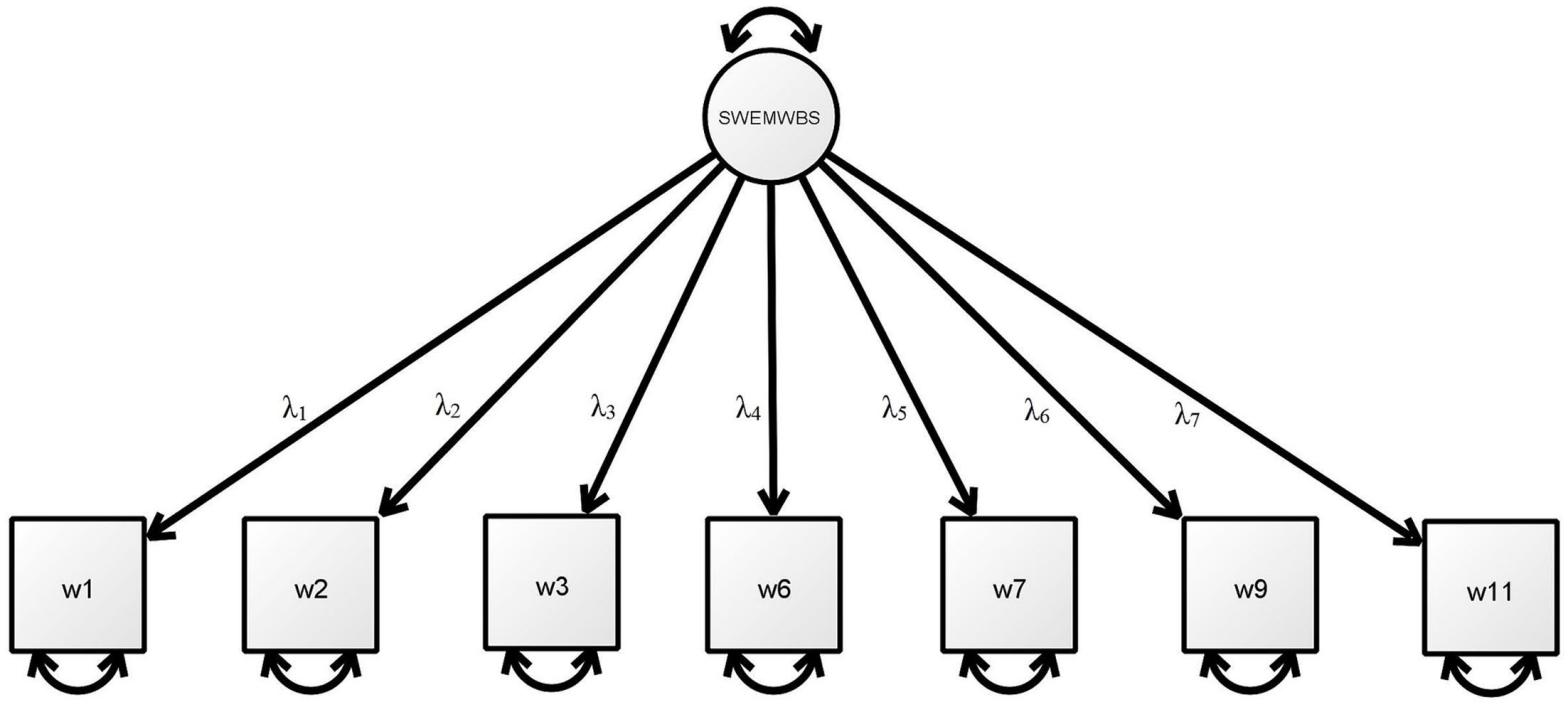
Estimated model of the 7-item SWEMWBS.

Confirmatory factor analysis was conducted to evaluate the construct validity of the SWEMWBS in both Traditional and Simplified Chinese versions (see Table 2 and Figure 1). The CFA results indicated that the Traditional Chinese version of the SWEMWBS possessed a good model fit, as *χ*^2^ (11.931)/14 = 0.85, SRMR = 0.038; CFI = 0.999; TLI = 0.999; and RMSEA = < 0.001 in Model 2. The results for Studies 1 and 2 combined (Combo 1) were similar: *χ*^2^ (14.224)/14 = 1.02, SRMR = 0.023, CFI = 0.999, TLI = 0.999, and RMSEA = 0.007.

**Table 2:** Factor loadings and fit indices in CFA for the SWEMWBS (see Figure 1 for estimated model).

Item		Study			
		2	3	4	Combo 1
w1	λ_1_	0.525	0.586^a^	0.835^a^	0.640
w2	λ_2_	0.642	0.702^a^	0.862^a^	0.772
w3	λ_3_	0.598	0.559	0.797	0.693
w6	λ_4_	0.535	0.673^b^	0.842^b^	0.693
w7	λ_5_	0.531	0.556^b, c^	0.815^b^	0.676
w9	λ_6_	0.598	0.608	0.804	0.664
w11	λ_7_	0.425	0.640^c^	0.832	0.658
Model fit					
*N*		183	511	887	331
RMSEA		< 0.001	0.044	0.045	0.007
RMSEA 90% CI		< 0.001–0.062	0.015–0.071	0.028–0.064	< 0.001–0.054
SRMR		0.038	0.028	0.017	0.023
*χ* ^2^		11.931	21.863	33.769	14.224
df		14	11	12	14
*χ*^2^/df		0.85	1.99	2.81	1.02
CFI		0.999	0.983	0.999	0.999
TLI		0.999	0.968	0.999	0.999

All factor loading *p* < 0.001; a–c: added error terms (items 1 with 2, 6 with 7 and 7 with 11); RMSEA, root mean square error of approximation; SRMR, standardised root mean residual; CFI, Comparative fit index; TLI, Tucker Lewis index. Combo 1 = Combined study 1 and 2.

The CFA results for the Simplified Chinese version of the SWEMWBS (Table 2, Models 3 and 4) also indicated a good model fit after *post hoc* modification. This procedure has been well justified in the existing SWEMWBS literature (Sarasjärvi et al., 2023; Smith et al., 2017). Based on the modification indices and recent findings related to the Simplified Chinese version of the SWEMWBS (Fung, 2019), we correlated the error terms between the items. The CFA results of Model 3, reported in Table 2, were *χ*^2^ (21.863)/11 = 1.99, SRMR = 0.028, CFI = 0.983, TLI = 0.968, and RMSEA = 0.028. Likewise, the Model 4 results were *χ*^2^ (33.769)/12 = 2.81, SRMR = 0.017, CFI = 0.999, TLI = 0.999, and RMSEA = 0.045. Overall, the CFA results indicated a good model fit for both the Traditional and Simplified Chinese versions of the SWEMWBS with a single factor structure.

### 3.3 Evaluation of the factor structure

In view of the recent controversies related to the factor structure of the SWEMWBS, we conducted bi-factor analysis (Sarasjärvi et al., 2023), with an additional latent factor structure of eudiamonic well-being (items 6, 7, and 11) based on the data from all four cross-sectional studies (*N* = 1,729). In Table 3, the CFA results show that only Studies 1 and 3 indicated good model fit and all the factor loadings were above 0.30. However, Studies 2, 4 and Combo 2 results did not fully satisfy the criteria for good model fit, as the factor loadings of item 6 (Model 2), 7 (Model 2), and 11 (Models 2, 4 and Combo 2) were below the acceptable range. The results suggest that the bi-factor structure only has good factorial validity among the samples from university students, i.e., participants from Studies 1 and 3.

**Table 3:** CFA models for the SWEMWBS with bi-factor structure.

Parameter	Study 1		Study 2		Study 3		Study 4		Combo 2	
	Mental well-being	Eudaimonic well-being	Mental well-being	Eudaimonic well-being	Mental well-being	Eudaimonic well-being	Mental well-being	Eudaimonic well-being	Mental well-being	Eudaimonic well-being
w1	0.766		0.521		0.683		0.871		0.837	
w2	0.848		0.633		0.789		0.898		0.877	
w3	0.778		0.591		0.567		0.805		0.781	
w6	0.759	0.343	0.550	**0.024** ^**#**^	0.583	0.465	0.807	0.433	0.801	0.371
w7	0.666	0.346	0.579	**−3.111** ^**#**^	0.499	0.564	0.783	0.422	0.774	0.382
w9	0.694		0.583		0.580		0.794		0.789	
w11	0.707	0.580	0.459	**0.047** ^**#**^	0.557	0.390	0.789	**0.234**	0.773	**0.255**
Model fit										
*N*	148		183		511		887		1,729	
RMSEA	0.020		< 0.001		0.035		0.035		0.032	
RMSEA 90% CI	< 0.001–0.090		< 0.001–0.059		< 0.001–0.064		0.014–0.055		0.019–0.046	
SRMR	0.025		0.031		0.025		0.014		0.012	
*χ* ^2^	11.676		8.040		18.045		23.088		30.873	
df	11		11		11		11		11	
*χ*^2^/df	1.06		0.73		1.64		2.10		2.81	
CFI	0.999		1.000		0.989		0.995		0.996	
TLI	0.997		1.000		0.980		0.991		0.993	

All factor loading *p* < 0.001, ^#^except; RMSEA, root mean square error of approximation; SRMR, standardised root mean residual; CFI, comparative fit index; TLI, Tucker Lewis index. Combo 2 = Combined Study 1, 2, 3 and 4. Bold text means the factor loadings below 0.30.

### 3.4 Criterion validity

Table 4 reports the correlation coefficients between the SWEMWBS with other construct-related measures used by the original WEBWBS developers to test its criterion validity (Tennant et al., 2007) from Study 3 (*n* = 511). The results show a significant moderate to strong positive correlation between the scale and PANAS - Positive Affect (*r* = 0.436, *p* < 0.001), WHO-5 (*r* = 0.537, *p* < 0.001), and SWLS (*r* = 0.484, *p* < 0.001). The results also supported the expectation that the SWEMWBS would display significant negative relationship with PANAS - Negative Affect (*r* = −0.243, *p* < 0.001). Hence, the results indicate good criterion validity for the SWEMWBS.

**Table 4:** Correlations between the SWEMWBS and other construct-related measures.

	SWEMWBS
PANAS - positive affect	0.436
PANAS - negative affect	−0.243
WHO-5	0.537
Satisfaction with life scale (SWLS)	0.484

All correlations are significant at the 0.001 level (2-tailed).

## 4 Discussion

Results indicate that both the Traditional and Simplified Chinese versions of the SWEMWBS possess unidimensional and good psychometric properties among secondary school students, university students, and working adults in different Chinese contexts. The coefficient alpha and omega values were above the acceptable, thus supporting the results of other SWEMWBS validation studies conducted in the United Kingdom, Spain, Norway, France, and India (Bartram et al., 2011; Castellví et al., 2014; Gremigni and Stewart-Brown, 2011; Ringdal et al., 2018; Stewart-Brown et al., 2009; Trousselard et al., 2016; Waqas et al., 2015). The EFA and CFA results also confirmed that the unidimensional SWEMWBS has an appropriate factor structure for mental well-being and the items can be combined to construct a scale to measure mental well-being.

The results of this study also indicated that the SWEMWBS possesses good criterion validity. The correlation findings largely replicated the magnitude and direction reported in existing WMEWBS literature, with PANAS - Positive Affect correlations ranging from *r* = 0.52 to 0.71, PANAS - Negative Affect from *r* = −0.25 to 0.54 (López et al., 2013; Tennant et al., 2007), WHO-5 from *r* = 0.46 to 0.77 (Dong et al., 2016; López et al., 2013; Taggart et al., 2013; Tennant et al., 2007), and SWLS from *r* = 0.55 to 0.71 (Lei et al., 2024; López et al., 2013; Nishida et al., 2016; Tennant et al., 2007; Vaingankar et al., 2017).

Regarding the dimensionality of the SWEMWBS, the results of the CFA (Table 3) reveal that only Studies 1 and 3 demonstrated an acceptable model fit, with all factor loadings exceeding 0.30 (Hair, 2010). In contrast, Studies 2, 4 and Combo 2 did not entirely meet the standards for a good model fit due to the factor loadings of items 6, 7, and 11 falling below the acceptable threshold. These findings indicate that the bi-factor structure exhibits solid factorial validity solely among the university student samples. The results of the bi-factor model replicate a recent study conducted in Finland, with CFI and TLI values of 0.995, SRMR of 0.013, and RMSEA of 0.063. The factor loadings for mental well-being and eudaimonic well-being range from 0.70 to 0.82 and 0.30 to 0.62, respectively (Sarasjärvi et al., 2023). In the Combo 2 (*N* = 1,729), the low factor loading (0.255) of item 11 raises questions about the existence of eudaimonic well-being as a latent factor structure in the SWEMWBS and calls for further investigation.

This study contributes to the application of the SWEMWBS in the following ways. First, this pioneering validation study used two versions of the SWEMWBS written in Traditional and Simplified Chinese, which are the commonly used languages in Chinese mainland, Hong Kong, Taiwan and other Chinese diaspora. Using the latest psychometric evaluation tools, the results of this study provide additional empirical evidence to support the use of the scale by researchers and medical practitioners to examine the mental well-being of the Chinese population. Second, they also provide supporting evidence that the SWEMWBS can be used in a female working population, which is an area that has rarely been explored in the previous WEMWBS studies in Chinese societies (Dong et al., 2016; Fung, 2019; Ng et al., 2014). Third, the current study examined the SWEMWBS in broader demographic of the Chinese population, including secondary school students, university students, and working populations. Previous studies examining the psychometric evaluation of WEMWBS have mainly focused on specific population groups, such as university students (Fung, 2019), patients with mental disorders (Ng et al., 2014), and individuals with chronic heart failure (Dong et al., 2019).

However, there are some potential limitations to the study. Sampling biases from using the convenience sampling method in Studies 1 and 3 may hinder the generalisability of the results. Additionally, the females recruited in Study 2 may not be representative of the target population, as they were sourced from specific organisations (e.g., a women’s organisation), and a criterion evaluation was not conducted, which could potentially be making generalisability problematic.

Furthermore, this paper is a consortium of multiple research projects in Hong Kong and Chinese mainland. Hence, the criterion validity of the SWEMWBS could not be evaluated in some contexts. To remedy the above limitations, this study incorporated a large sample of 1,729 participants with different demographic backgrounds in Hong Kong and Chinese mainland to evaluate the internal consistency, factorial validity, and construct validity of the scale. The nature of the descriptive, cross-sectional design adopted by this study hindered the assessment of the questionnaire’s responsiveness. Therefore, it is suggested that further evidence regarding the responsiveness of the SWEMWBS be sought in an appropriate longitudinal study. Despite these limitations, the results of this study replicated the findings reported in the SWEMWBS literature (Fung, 2019; Ng et al., 2014; Stewart-Brown et al., 2009).

## 5 Conclusion

In conclusion, this study critically evaluated the bi-factor structure of the Traditional and Simplified Chinese versions of the SWEMWBS using the latest psychometric evaluation tools. The results indicated that both Chinese versions of the SWEMWBS are unidimensional and possess good psychometric properties comparable to the original scale. The Chinese versions of the SWEMWBS allow for efficient and valid assessment of mental well-being for secondary school students, university students, and female working adults. These measures could be used in future studies to conduct epidemiological surveys or to evaluate the effectiveness of intervention programmes for the Chinese diaspora.

## Data Availability

The raw data supporting the conclusions of this article will be made available by the authors, without undue reservation.
